# Drug-microbiota interactions: an emerging priority for precision medicine

**DOI:** 10.1038/s41392-023-01619-w

**Published:** 2023-10-09

**Authors:** Qing Zhao, Yao Chen, Weihua Huang, Honghao Zhou, Wei Zhang

**Affiliations:** 1 Department of Clinical Pharmacology, Xiangya Hospital, Central South University, 87 Xiangya Road, Changsha, 410008 PR China; 2 Institute of Clinical Pharmacology, Central South University, Hunan Key Laboratory of Pharmacogenetics, 110 Xiangya Road, Changsha, 410078 PR China; 3 Engineering Research Center of Applied Technology of Pharmacogenomics, Ministry of Education, 110 Xiangya Road, Changsha, 410078 PR China; 4 National Clinical Research Center for Geriatric Disorders, 87 Xiangya Road, Changsha, 410008 PR China; 5 The First Affiliated Hospital of Shantou University Medical College, Shantou, 515041 PR China; 6 The First Affiliated Hospital of Guangdong Pharmaceutical University, Guangzhou, 510080 PR China; 7 Central Laboratory of Hunan Cancer Hospital, Central South University, 283 Tongzipo Road, Changsha, 410013 PR China

**Keywords:** Molecular medicine, Microbiology, Genetics research, Drug delivery, Molecular biology

## Abstract

Individual variability in drug response (IVDR) can be a major cause of adverse drug reactions (ADRs) and prolonged therapy, resulting in a substantial health and economic burden. Despite extensive research in pharmacogenomics regarding the impact of individual genetic background on pharmacokinetics (PK) and pharmacodynamics (PD), genetic diversity explains only a limited proportion of IVDR. The role of gut microbiota, also known as the second genome, and its metabolites in modulating therapeutic outcomes in human diseases have been highlighted by recent studies. Consequently, the burgeoning field of pharmacomicrobiomics aims to explore the correlation between microbiota variation and IVDR or ADRs. This review presents an up-to-date overview of the intricate interactions between gut microbiota and classical therapeutic agents for human systemic diseases, including cancer, cardiovascular diseases (CVDs), endocrine diseases, and others. We summarise how microbiota, directly and indirectly, modify the absorption, distribution, metabolism, and excretion (ADME) of drugs. Conversely, drugs can also modulate the composition and function of gut microbiota, leading to changes in microbial metabolism and immune response. We also discuss the practical challenges, strategies, and opportunities in this field, emphasizing the critical need to develop an innovative approach to multi-omics, integrate various data types, including human and microbiota genomic data, as well as translate lab data into clinical practice. To sum up, pharmacomicrobiomics represents a promising avenue to address IVDR and improve patient outcomes, and further research in this field is imperative to unlock its full potential for precision medicine.

## Introduction

Why does a drug with a well-response for one patient, but not for the next, or in some cases, lead to serious adverse drug reactions (ADRs), namely individual variability in drug response (IVDR)? The answers are complicated and multi-faceted. First of all, therapeutic response rates of different drugs caused by IVDR differ. According to a survey, cyclooxygenase-2 (COX-2) inhibitors exhibit the most remarkable response rate (80%) among drugs used to treat various diseases, while tumor chemotherapy displays the lowest response rate (25%).^1^ Furthermore, the response rates for various other medications varied from 50–75%, indicating that a significant proportion of patients did not benefit from these treatments.^1^ Secondly, the rate of ADRs caused by IVDR is not the same thing. ADRs are the joint occurrence and can have a substantial impact on morbidity, mortality, and healthcare costs.^2–5^ For example, ADRs accounted for approximately 4.93% of emergency hospitalizations in China, with a majority of them being preventable (around 73.7%).^6^ However, IVDR makes the prevention of ADRs tricky. These facts underscore the urgent need to better understand IVDR to meet the highly effective drug or prevent ADRs.

Precision medicine aims to develop tailored medical interventions on the basis of an individual’s genetic, environmental, and lifestyle elements.^7–9^ However, IVDR is a major barrier to the implementation of precision medicine, as it poses a challenge to predict drug efficacy and identify patients who are at risk for ADRs.^10–13^ Consequently, it is crucial to develop improved biomarkers that can predict drug efficacy and toxicity in order to successfully implement precision medicine.

Pharmacogenetics focuses on identifying genetic variants that influence drug metabolism, transport, pharmacodynamics (PD), and others.^14–19^ Pharmacogenomics is a broader field that considers whole genome and its impact on drug response.^11,13,20–26^ Despite the progress made in pharmacogenetics and pharmacogenomics, studies to determine IVDR have shown that human genome contributes anywhere between 20% and 95% of the variability (this percentage may vary depending on the disease model).^27^ Hence, genetic factors alone are insufficient to explain IVDR, and additional factors (such as diversity in gut microbiota spectrum) may also be important.^28–34^

Gut microbiota, which is a bifunctional and heterogeneous ecosystem, often described as a ‘metabolic organ’, contains over 100 trillion microbes and 5 million genes, making it much larger than human gene count (~150 times).^35–45^ Due to various host-related factors, gut microbiota composition and structure vary greatly between individuals and over time as well as external factors (e.g., drugs, diet, and environment).^46–52^ Studies conducted on both mice and humans have demonstrated that drug intake has significant effects on the structure of gut microbiota.^53–55^ In recent years, gut microbiota has also been mandated for IVDR, gut microbiota can alter drug PD and pharmacokinetics (PK) by direct transforming the drug or modulating the metabolism or immune system of the host.^42,54,56–58^

The term pharmacomicrobiomics has been coined to describe the interactions between gut microbiota and drug response that can alter PK (i.e., changes in drug absorption, distribution, metabolism, excretion, and its plasma drug concentration dynamics) or PD (i.e., changes in drug targets or biological pathways resulting in differential susceptibility of the organism to pharmacological effects).^59–65^ Understanding this interaction can help develop microbiota-targeted approaches to enhance drug efficacy and reduce ADRs.^66–68^ Clearly, pharmacomicrobiomics is becoming an integral part of the advances in precision medicine, and modifying gut microbiota could be a highly appealing option for managing the efficiency and safety of drugs at an individualistic level.^58,61^ This review outlines pharmacomicrobiomics advances in human diseases to better understand the impact of IVDR in future precision medicine.

## Background

### Causes of the individual variability in drug response (IVDR)

**Fig. 1:**
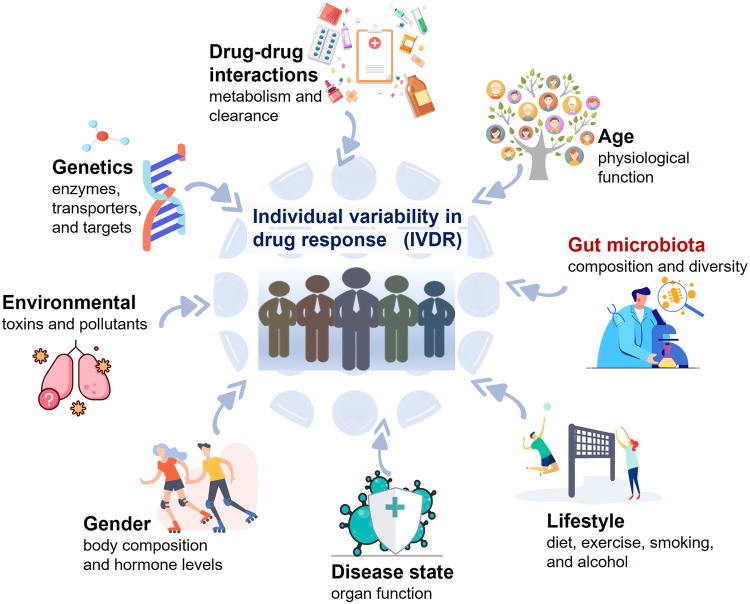
Causes of the individual variability in drug response (IVDR)

IVDR is a complex and multifactorial phenomenon that can result from a combination of factors, as shown in Fig. 1, including (1) Genetics: Variations in individual genes encoding drug-metabolizing enzymes, transporters, and targets can affect their metabolism and response to drugs, leading to IVDR.^69–73^ (2) Age: Younger and older patients may experience different drug responses due to differences in their physiological function, such as changes in liver and kidney function, body composition, and hormone levels.^74–78^ For example, elderly patients may have reduced renal function and hepatic metabolism, leading to altered PK.^79–81^ (3) Gender: Biological dissimilarities between men and women can influence drug response.^82–87^ Women may have higher drug concentrations due to disparities in body composition and hormone levels, which will affect drug metabolism and clearance.^88–91^ (4) Lifestyle: Factors such as diet, exercise, smoking, and alcohol intake can affect drug response by changing the way drugs are metabolized and eliminated from the body.^92–95^ (5) Disease state: Patients with certain diseases or conditions may experience IVDR due to changes in organ function, altered drug metabolism, or altered drug receptor expression.^96–99^ For example, drug metabolism may be reduced in patients with liver diseases and drug clearance may be reduced in patients with kidney diseases.^100–103^ (6) Drug-drug interactions: Concomitant use of multiple medications may result in drug-drug interactions that alter drug absorption, distribution, metabolism, and excretion (ADME).^104–107^ (7) Environmental factors: Exposure to environmental toxins and pollutants can affect drug metabolism and clearance, potentially leading to IVDR.^108–112^ (8) Gut microbiota: Gut microbiota may also affect drug PK and PD, and variations in the composition of gut microbiota between individuals contribute to IVDR.^42,56,57,66,113–115^ Overall, understanding these factors is critical to optimizing drug therapy and minimising the risk of ADRs.

#### Heterogeneity of the human genome profile in IVDR

In the early 1900s, Archibald Garrod, a renowned physiologist from England, proposed that genetic factors may contribute to inborn errors in drug metabolism and IVDR.^116,117^ Since then, numerous studies have demonstrated that genetic factors can explain as much as 95% of the inter-individual variability in drug PK and PD for specific drugs or drug classes^118–121^ (Table 1). In the late 1950s, it was discovered that inherited glucose-6-phosphate dehydrogenase deficiency could lead to serious hemolysis in patients administered primaquine, an anti-malarial drug, primarily in African-Americans and minimally in Caucasians of North, West, and East European heritage.^122–124^ Subsequently, in 1959, Vogel introduced the term pharmacogenetics to define IVDR.^125^ The evolution (from genetics to genomics) and application of genome-wide strategies has led to the emergence of the term pharmacogenomics, with the potential to target specific drug therapies to genetically defined patient subpopulations and to establish novel diseases and therapeutic classifications at the molecular and phenotypic level.^15,20–22,126–128^

**Table 1:** Comparison of pharmacogenomics and pharmacomicrobiomics in IVDR

Individual variability	Pharmacogenomics	Pharmacomicrobiomics
Dependent variables	Pharmacokinetic (PK) and pharmacodynamic (PD) outcomes.	
Independent variables	Variations in the human genome that are inherited from generation to generation.	Composition and structure of gut microbiota, and its genomic, metabolomic, transcriptomic, and proteomic variations.
Intra-individual variations	The human genome is a relatively stable entity, with only rare mutations emerging during an individual’s lifetime.	Human microbiota is a dynamic and mobile entity, similar to a cloud, whose composition and structure are constantly changing under the influence of intra-individual variations (e.g., hormones in vivo), leading to subtle and constant changes in physiology.
Inter-individual variations	Pharmacogenetics and pharmacogenomics are the study of genetic and genomic variations that influence drug response inter-individuals IVDR. Allelic variations in drug-metabolizing enzymes, such as cytochromes, are among the most commonly studied. Other allelic variations that may affect drug action include transporters, and target molecules or receptors.	Increasing attention has been focused on functional rather than microbiota profile-based classifications of inter-individual variations. By this means, different functional clusters or metabolic phenotypes have been identified between individuals, which can provide valuable information for insight into the underlying mechanisms of health and disease. The potential of single biomarkers (e.g., *Bifidobacterium*) as tools to assess health and disease between individuals.

IVDR poses a serious challenge in clinical management, and genetic polymorphisms play a vital role in this variability.^16,17,25^ Genetic variations in genes associated with drug transport, metabolism, and targets can change the individual’s sensitivity to treatment, resulting in variable drug response.^121,129–137^ The function of transporters in controlling the transfer of drugs and their metabolites into and out of cells is critical to drug efficacy and toxicity.^138,139^ In the case of pro-drugs, metabolism is essential to prevent ADRs caused by elevated plasma drug levels in some patients. However, environmental factors can also influence drug metabolism, efficacy, and toxicity, leading to IVDR.^18,140^ Despite the important influences of genetic variation on drug response, the complexity of environment and microbiota may also constrain the prediction of drug response on the basis of genomic diversity alone.^141^ Hence, it is crucial to have a comprehensive comprehension of the intricate interaction between genetic and external factors to develop precision medicine strategies that optimize drug therapy for individual patients.

IVDR can also be attributed to the presence of variant alleles of drug-metabolizing enzymes, transporters, and targets in different populations.^142–144^ Allelic variation of target genes in the study population is essential for the advancement of personalized tumor treatment.^145–150^ However, the frequency of unique variant alleles can vary significantly between races, making it difficult to incorporate pedigree into clinical decision-making because it confounds drug response.^25^ In addition, PK differences between drug-metabolizing enzyme and transporter populations can affect IVDR and raise the risk of ADRs (e.g., thiopurines, allopurinol, and carbamazepine).^143,151^ A lot of ADRs are linked to specific phenotypes of drug metabolites, such as hypersensitivity reactions triggered by sulfonamides.^25,152–154^ A more in-depth mechanistic and phenotypic study of drug metabolism and individual differences between patients may help to guide treatment based on unique characteristics and the application of predictive models to avoid ADRs.^25^

#### Diversity of microbiota spectrum in IVDR

**Fig. 2:**
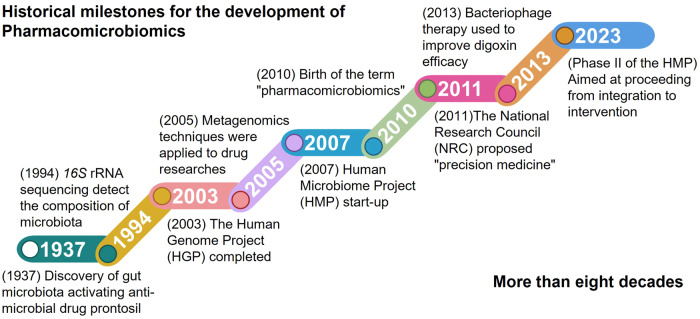
Timeline of the historical milestone for the development of pharmacomicrobiomics in the past eight decades

The Human Genome Project (HGP) was concluded in 2003, but human genome contains fewer coding genes, and variations in genetic, epigenetic, and regulators are insufficient to describe IVDR in the phenotype, with limited utility for precision medicine^155^ (Fig. 2). The focus has shifted to gut microbiota to study its composition, variation, and function in understanding the spectrum of human phenotypic variation, including its impact on well-being, immune system, and drug response. In comparison to human genome, gut microbiota is remarkably adaptive and mobile, similar to a cloud whose components and genetic pool are unclear at every moment in time and space^156^ (Table 1). It can be partially or completely swapped out and is exposed to various factors that influence its evolution, including immune responses, phage attacks, diet, toxins, anti-biotics, and others.^157,158^ Because of the abilities of gut microbiota to influence drug response and disease progression, targeted manipulation of gut microbiota may improve drug efficacy and reduce drug-drug interactions.^141^ Mobility of gut microbiota is not limited to movement within inter-individuals, but can also occur within intra-individuals due to factors such as spatial, transient, seasonal, hormonal, and nutritional changes or the presence of multiple drugs.^159^ The diversity of gut microbiota gives it a remarkable metabolic capacity that exceeds even that of the host.^160–162^ Specifically, gut microbiota can produce a range of metabolic responses to drugs and xenobiotics, resulting in both direct effects on drug metabolism and toxicity, and indirect effects on host metabolic enzymes/transporters/immune system, these responses can ultimately affect IVDR.^45,163^ Gut microbiota can directly affect drug metabolism in several manners, including generating enzymes that degrade or catalyze a drug molecule, competing with drug molecules for a metabolic enzyme, altering the metabolic levels of drugs in the host, or generating enzymes that stimulate metabolites originally derived from the diet.^59,164^ The presence of microbiota-encoded enzymes represents a potentially valuable intermediate target for PK modification that may ultimately improve clinical response.^44^ In turn, drug administration may also affect microbiota metabolism or growth, leading to variations in the structure and function of microbiota.^63^ Changes in a pathological state, metabolic enzyme expression, and drug transporter expression may result in gut microbiota producing compounds that affect drug PK and PD.^165^

In addition, human genome and gut microbiota work in tandem to influence personal metabolism and contribute to IVDR.^59^ Through anatomical and physiological linkages (e.g., the intestinal barrier), they exchange metabolically active molecules and beneficial host-immune interactions.^164,166^ This metabolic axis of gut microbiota links human genome to microbiota ecosystem as a genetic or epigenetic basis for up-keeping human immune and nutrition functions.^166–169^ Interactions between human genome and the diversity of gut microbiota genomes lead to the synthesis of compounds benefiting human healthcare, e.g., amino acids (AAs), short-chain fatty acids (SCFAs), and bile acids (BAs).^165,170^ Genes encoded by gut microbiota greatly enhance the metabolic potential of human, contributing as much as 36% of small molecule metabolism in human blood.^59^ Therefore, the measurement of large numbers of metabolites is necessary to better report and manage the multitude of diseases and treatment efficacy issues.^171^

In summary, understanding the influence of human genome heterogeneity and gut microbiota spectrum diversity on IVDR is a key component in developing and improving precision medicine while minimizing ADRs.^59^ In addition, modeling changes in gut microbiota will help to understand xenobiotic metabolism, thereby accurately predicting drug responses by taking into account genetic diversity and metabolic interactions between host and pertinent microorganisms.^59^

### Human microbiome exploration in IVDR

The microbiome encompasses the genetic material of various microorganisms, such as bacteria, fungi, protozoa, and viruses, that inhabit both the external and internal regions of the host.^172–174^ The term microbiome can denote the genetic make-up of the microbiota or the entire genome of all its members.^59,175,176^ Microbiome can also refer to the total environment that microorganisms inhabit.^177^ Meanwhile, microbiomics is a scientific discipline that utilizes high-throughput molecular approaches to study microbial communities.^178–180^

The Human Microbiome Project (HMP) is a collaborative program initiated by the National Institutes of Health (NIH) in 2007 to explore in depth the diversity and functional of the human microbiota^181^ (Fig. 2). The project aims to apply high-throughput sequence techniques to characterize microbiota from multiple body sites in human, to investigate the importance of microbiota in IVDR, and to initiate novel research tools and resources for the scientific community. The HMP involved a consortium of more than 200 researchers across multiple institutions, whose findings greatly enhance our knowledge of microbiota and its impact on human health and IVDR. The project was completed in 2012, and its data and resources continue to be used in ongoing microbiota research. The second phase of the HMP started in 2014 and is still in progress.^182^ With the support of the HMP, numerous revolutionary findings have significantly enhanced our understanding of microbiota-associated disorders. The discovery of these results has led researchers to reevaluate the origin and development of human diseases and utilize the unique capabilities of the microbiota to create innovative diagnostic methods and more accurate treatments. Furthermore, numerous projects such as the Earth Microbiome Project (EMP), Integrative Human Microbiome Plan (iHMP), and Metagenomics of the Human Intestinal Gut (MetaHIT) have been initiated to further analyze the composition and functions of microbiota, resulting in the accumulation of extensive microbiota data represented as phylogenetic or functional composition profiles.^41,183–185^ Meanwhile, it has also facilitated the emergence and development of new microbiota-related fields of discipline.

## Emerging of pharmacomicrobiomics in IVDR

**Fig. 3:**
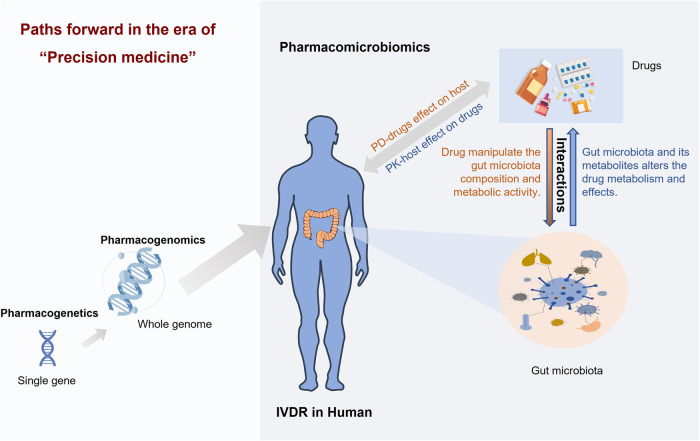
An overview of pharmacomicrobiomics is a rapidly growing field that seeks to understand the complex interactions between drugs, microbiota, and host physiology

Recently, the research of drug-microbiota interactions has become a systematically developed field, due to the increasing fascination with the variation of gut microbiota.^62,186–189^ This field has given rise to various sub-disciplines, including pharmacomicrobiomics, pharmacometabonomics, and pharmacometagenomics.^159,190–193^ These sub-disciplines aim to study the effect of microbiota on PK and PD, and to explore how microbiota variation affects IVDR in human diseases^194–197^ (Fig. 3). In addition, these areas explore systems-level studies of microbial metabolites and their diagnostic and implications for pharmacological properties of drugs.^57,198,199^

In fact, during the 1930s, several research teams reported that prontosil had a beneficial effect on mice infections of various strains. But the outcomes were considered atypical regarding the lack of anti-bacterial activity of prontosil in vitro^200^ (Fig. 2). Nevertheless, sulfanilamide, a metabolite of prontosil, exhibited potent anti-bacterial effects in vivo, suggesting that the anti-bacterial properties of prontosil stemmed from this derivative.^200,201^ Subsequently, numerous sulfonamide compounds have been synthesized and used to treat bacterial infections, and many studies of drug-microbiota interactions have sprouted among them. Until 2010 when it was first proposed, the term ‘pharmacomicrobiomics’ was used to describe the effects of alterations in microbiota composition and function on IVDR and host genetic^190^ (Fig. 3). While the interactions between drugs and individual microbiota have been studied for some time, advanced and accurate meta-omics technologies, including *16* *S* ribosomal RNA (rRNA) amplicon, *18* *S* rRNA amplicon, *ITS*, *MiSeq*, *HiSeq*, and whole-metagenome shotgun sequencing have facilitated a deeper exploration into the phylogeny, interactions, and functions of microbiota species.^202–206^ By using these technologies, the development of pharmacomicrobiomics has made significant progress, providing a comprehensive analysis of the connection between drugs and the microbiota.

To study the effects of gut microbiota on drugs, terms such as pharmacometabolomics and pharmacometabonomics can be used in addition to pharmacomicrobiomics.^141,191,207,208^ These fields focus on the systems-level study of metabolites from microbiota, integrating genomics and metabolomics.^198,209–212^ While primarily concerned with microbiota variation, these fields also study the ultimate metabolic products of host-microbiota interactions, namely the metabolites that are generated as a result of drug metabolizing in humans and with its co-occurring microbiota.^208,213^

Pharmacometagenomics is another term used to study IVDR by integrating the analysis of microbiota genomes and human genetics.^193^ While metagenomics studies the species and gene pool of ecosystems, pharmacometagenomics specifically examines shotgun sequencing or chance microbiota genome analysis, and it needs to be distinguished from amplicon analysis, which merely determines the phylogenetic tree of the living beings. What characterizes pharmacometagenomics is that it integrates microbiota composition (by *16* *S* or *18* *S* amplicon sequencing) and potential function (by metagenomics, metaproteomics, metatranscriptomics, and metabolomics), all elements of microbiomics. The integration of multi-omic approaches is essential, as it can be difficult to solely predict function from microbiota.^214^

**Fig. 4:**
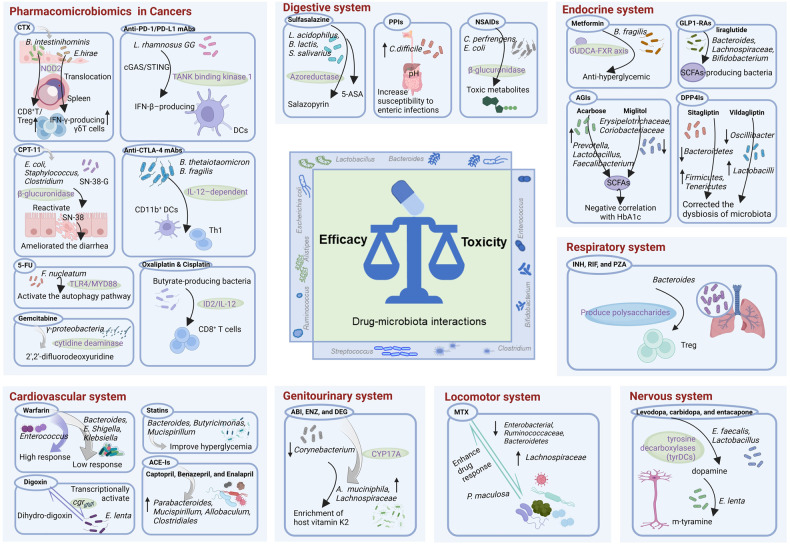
Examples of drug-microbiota interactions with well-defined mechanisms. Created with BioRender.com

The emerging field connecting the composition and activity of microbiota to prevalent diseases and drugs has spawned numerous promising discoveries in recent years. Association studies have revealed bidirectional effects between various drugs and gut microbiota in patients with gastrointestinal disorders (e.g., inflammatory bowel diseases and colorectal cancer) and other system diseases (e.g., cardiovascular and metabolism disorders, autoimmunity diseases, and psychiatric disorders). These findings have important implications for understanding IVDR and developing precision medicine approaches tailored to individual microbiota composition and function. We will discuss these findings in greater detail below (Fig. 4).

## Pharmacomicrobiomics of IVDR in human diseases

### Pharmacomicrobiomics in cancers

In the context of cancer, gut microbiota is known to be critical for the pathogenesis, progression and IVDR of several types of cancers.^215,216^ Numerous studies have investigated the potential of pharmacomicrobiomics to improve tumor treatment outcomes (Table 2). Here, we describe the mechanisms by which several representative drugs and gut microbiota interact in cancer patients.

**Table 2:** Examples of immunotherapies and chemotherapies interactions with the gut microbiota

Drugs		Involved bacteria	Mechanisms of interaction with microbiota	Animal Models	PMID
Chemotherapies	Cyclophosphamide (CTX)	*Escherichia coli, Enterobacteraceae, Pseudomonas*, and *Enterococci*	Increased intestinal permeability due to decreased function of tight junctions and adhesive junctions.	Male Balb/c mice.	23791611
		*Bacteroides acidifaciencs* and *Streptococcaceae*	Increased bacteria.	C57/BL6 mice.	25553830
		*Lactobacillus johnsonii* and *Enterococcus hirae*	Increased Th17-mediated IL-17 secretion. From the gut to the secondary lymphatic organs is essential for the differentiation of CD4^+^ T cells.	B16F10 melanoma mouse.	24264990
		*Enterococcus hirae* and *Barnesiella intestinihominis*	*E. hirae* increased the CD8^+^ T/Treg cells ratio within tumors, *B. intestinihominis* promoted the infiltration of IFN-γ-producing γδT cells into tumors.	MCA205 sarcoma mice.	27717798
	Irinotecan (CPT-11)	*Escherichia coli, Staphylococcus*, and *Clostridium*	Express high levels of β-glucuronidase, a microbial enzyme that eliminates SN-38-G.	Rats.	18927500
		*Lactobacillus* and *Bifidobacterium*	Reducing β-glucuronidase activity and diarrhea.	C57/BL6 mice.	33298215
		*Enterococcus faecium*	Reducing β-glucuronidase activity and diarrhea.	Rats.	8706020
	5-Fluorouracil (5-FU)	*Fusobacterium nucleatum*	Targeting the TLR4-MYD88 pathway and activating the autophagy pathway.	HCT116 xenograft mouse.	28753429
		*Bacteroides (B. vulgatus, B. thetaiotaomicron, B. fragilis, B. uniformis*, and *B. eggerthii)*	Produces uracil metabolites that inactivate dihydropyrimidine dehydrogenase and cause toxicity.	Male SD rats.	9110360
	Gemcitabine	*γ-proteobacteria*	Bacterial long isoform cytidine deaminase metabolism gemcitabine into inactivated form 2′,2′-difluorodeoxyuridine.	MC-26 xenograft mouse.	28912244
		*Mycoplasma hyorhinis*	Encoded cytidine deaminase.	Human breast carcinoma MDA-MB-231 cells and MCF-7.	26322268
	Oxaliplatin & Cisplatin	Gram-negative bacteria	Regulate the TLR4-dependent pathway to prepare myeloid cells for ROS release.	EL4, MC38, and B16 xenograft mouse.	24264989
		*Bacteroides fragilis* and *Erysipelotrichaceae*	Induce migrating DCs to transduce IL-1β and IL-12 responses to follicular Th cells.	MC38 and CT26 xenograft mouse.	32451498
		Butyrate-producing bacteria	Butyrate activated CD8^+^ T cells via ID2-dependent IL-12.	Male Wistar rats.	33761313
Immunotherapies	Anti-CTLA-4 mAbs	*Bacteroides fragilis, Bacteroides thetaiotaomicron*, and *Burkholderia cepacia*	Stimulating CD11b^+^ DCs improves IL-12-dependent Th1 immunity.	MCA205 sarcoma mice.	26541610
		*Bifidobacterium*	Enhanced functions of intestinal Treg cells.	MCA205 sarcoma mice.	33077598
		*Bifidobacterium pseudolongum, Lactobacillus johnsonii*, and *Olsenella species*	Enhanced immune response through producing inosine.	C57BL/6 and Foxp3-DTR mice.	32792462
	Anti-PD-1/PD-L1 mAbs	*Bifidobacterium*	Promoting the role of DCs and activating cytotoxic CD8^+^ T cells.	B16.SIY melanoma mouse.	26541606
		*Bifidobacterium*	Increased gut permeability leading to intratumor localization, enhanced NK cell functions.	B16F10 and LLC melanoma mouse.	34516769
		*Bifidobacterium breve*	Bacterial antigens affect the T cells.	SIY melanoma mouse.	32324171
		*Lactobacillus rhamnosus* GG	Stimulatiing cGAS/STING/TANK binding kinase 1/interferon regulatory factor 7 axis in DCs.	MC38 and B16F10 xenograft mouse.	33685966

#### Cyclophosphamide (CTX)

CTX is a pro-drug that needs activation, with its primary active ingredient being phosphoramide mustard, which creates crosslinks in inter and intra-strand deoxyribonucleic acid (DNA).^217–220^ T cells, particularly cytotoxic T cells, play a crucial role in targeting tumor cells in various cancers, and increased T cell infiltration into tumors has been linked to better patient outcomes.^221–224^ Previous microbiota studies have identified specific microbiota associated with improved anti-tumor T cell responses following tumor therapy.^225^ CTX activates anti-tumor immune response, thereby boosting the differentiation of anti-tumor T helper (Th)-1 and Th17 cells, exhausting pro-tumor regulatory T (Treg) cell populations, stimulating the secretion of various cytokines, and increasing the death of immunogenic tumor cells.^226–233^ Saxman et al. compared the efficacy of combined CTX with CTX alone and showed IVDR of 18.8% in the combined group compared with 6% in the other group.^234^ It is worth noting that CTX treatment results in increased intestinal permeability due to decreased function of tight junctions and adhesive junctions.^235,236^ This increased permeability is associated with higher levels of pathogenic strains, e.g., *Escherichia coli* (*E. coli*)*, Enterobacteraceae, Pseudomonas*, and *Enterococci* in the gut.^236^ Furthermore, CTX treatment reduces fecal microbiota diversity and increases the *Firmicutes*-to-*Bacteroidetes* ratio.^237^ Several species and families were found in CTX-treated samples but not in untreated samples, e.g., *Bacteroides acidifaciencs* and *Streptococcaceae*, even though the implications of this are not well known.^237^

A study conducted in 2013 discovered that germ-free (GF) and antibiotic-treated mice had a noticeably decrease anti-tumor response to CTX treatment.^235^ In particular, CTX treatment resulted in increased Th17-mediated pro-inflammatory interleukin (IL) -17 secretion in specific pathogen-free (SPF) mice, but not in GF or antibiotic-treated mice. This research further revealed that the CTX-mediated translocation of gram-positive bacteria, e.g., *Lactobacillus johnsonii* and *Enterococcus hirae* (*E. hirae*), from the gut to the secondary lymphatic organs is vital to differentiation of CD4^+^ T cells.^235^ Co-administration of CTX and vancomycin had worse total anti-tumor activity than CTX monotherapy, emphasizing how essential elements of the microbiota influence the action of CTX.^235^ Further research has identified *E. hirae*, when translocated from the gut to the secondary lymphatic organs, increased the CD8^+^ T/Treg cells ratio within tumors.^238^ On the other hand, *Barnesiella intestinihominis* does not translocate but accumulates in the colon, stimulating CD8^+^ T cells and Th1 cells, thus enhancing the infiltration of interferon (IFN)-γ-producing γδT cells into tumors.^238^ The interaction between IVDR of CTX and the microbiota is complex, as CTX affects the intestinal barrier integrity, resulting in the translocation of pathogenic species, while being affected by commensal bacteria for pharmacological immune-mediated efficacy. Such discoveries emphasize the significance of considering gut microbiota in the use of CTX and other chemotherapeutic agents.

#### Irinotecan (CPT-11)

Chemotherapeutic agents such as CPT-11 are commonly for treating several cancers, however, their use can be limited by the occurrence of severe diarrhea as ADRs.^239–242^ This side effect can be attributed to the influence of gut microbiota, which can convert the less toxic SN-38-glucuronide (SN-38-G) metabolite back into the extremely toxic 7-ethyl-10-hydroxycamptothecin (SN-38), leading to intestinal damage.^243,244^ Specifically, certain strains (e.g., *E. coli, Staphylococcus*, and *Clostridium*) that express high levels of β-glucuronidase (GUSs), a microbial enzyme that eliminates SN-38-G, have been shown to exacerbate this problem.^245^ However, antibiotic administration to decrease the abundance of such strains has been investigated as a potential strategy to alleviate CPT-11-induced diarrhea.^246,247^

In clinical trials, the use of anti-biotics (e.g., levofloxacin, cholestyramine, and neomycin) has been evaluated in conjunction with CPT-11 treatment.^246,247^ As a result, the overall incidence of IVDR was significantly decreased from the 40% reported in earlier studies.^246^ Administering neomycin alongside CPT-11 reduced or eliminated diarrhea in six out of seven volunteers, according to another study.^247^ Although co-administration of these drugs leads to a reduction or elimination of diarrhea, concerns have been raised about the possible consequences for gut microbiota and the emergence of antibiotic resistance.^248^ Additionally, selective inhibition of microbial GUSs has been investigated as an alternative strategy.^249,250^ Repurposing existing drugs and designing new GUSs inhibitors have been explored, with promising results demonstrating effective reduction of diarrhea without compromising the anti-tumor effects of CPT-11.^249,251–253^

The administration of probiotics another potential strategy for mitigating CPT-11-induced diarrhea has also been investigated. *Enterococcus faecium* (*E. faecium*) and multi-strain probiotics (e.g., *Lactobacillus* and *Bifidobacterium*) have shown effectiveness in reducing GUSs activity and diarrhea in clinical studies.^254,255^ However, the potential risks and costs of probiotics must be carefully considered, particularly when administering them to immunocompromised individuals, as they could potentially increase the risk of superinfection.

In conclusion, the intervention of gut microbiota has shown potential in mitigating the ADRs associated with CPT-11 treatment. While the use of anti-biotics and probiotics has proven effective in reducing diarrhea, their potential modulation of gut microbiota and the occurrence of anti-biotic resistance must be carefully considered. Selective inhibition of microbial GUSs offers a promising alternative strategy to mitigate CPT-11-induced diarrhea. More in-depth studies are warranted to assess the effects and risks of such measures in larger patient populations.

#### Oxaliplatin and cisplatin

Platinum-containing anti-neoplastic drugs, including oxaliplatin and cisplatin, have been used extensively to treat several cancers.^256–258^ These drugs work by interfering with the DNA replication process in tumor cells, leading to their death.^259–261^ However, the effectiveness of these drugs relies on the generation of reactive oxygen species (ROS), which cause oxidative injury to tumor cells. Interestingly, recent reports reveal that gut microbiota is crucial for the production of ROS and IVDR associated with these drugs.^262,263^ Specifically, gut microbiota, particularly gram-negative bacteria, regulate the myeloid differentiation primary response 88 (MYD88) related pathway to prepare myeloid cells for ROS release in the tumor undergoing treatment with oxaliplatin.^263^ This means that the integrity of the microbiota is necessary for the early cytotoxic effects of these drugs. Moreover, gut microbiota also plays a significant role in the late immunomodulatory effects of oxaliplatin.

For an effective anti-cancer immune response, immunogenic gut commensals are required, along with the antigenic properties of the apoptosis mediated by oxaliplatin. Immunogenic bacteria, such as non-enterotoxigenic *Bacteroides fragilis* and *Erysipelotrichaceae*, induce migrating dendritic cells (DCs) to transduce IL-1β and IL-12 responses to follicular Th cells.^264^ This interaction leads to increased IgG2b reaction and increased anti-cancer effector CD8^+^ T cells activity. It is important to mention that without immunogenic gut commensals, these immune responses are greatly diminished.

Moreover, metabolites such as SCFAs can also help to the improvement of the immune response to oxaliplatin.^265^ SCFAs, especially butyrate, are able to block histone deacetylases (HDCA) to upregulate the DNA transcription modulator inhibitor of DNA binding 2 (ID2).^266^ This, in turn, enhances the cytotoxic activity of CD8^+^ T cells through IL-12 signaling, promoting anti-tumor immunity generated by oxaliplatin. In conclusion, gut microbiota and their metabolites are major regulators of the anti-tumor immunity responses to platinum-based drugs.

#### Immune checkpoint inhibitors (ICIs)

ICIs have revolutionized tumor therapy by specifically modulating inhibitory receptors (e.g., CTLA-4, PD-1, LAG-3, and TIM-3) and ligands (PD-L1) presented on immune and tumor cells, ultimately stimulating anti-tumor IVDR.^267–269^ Emerging preclinical evidence also emphasizes the role of gut microbiota in modulating the IVDR of ICIs treatment by influencing the immune response.^235,238,263^

Studies using preclinical mouse models have shown that an effective anti-tumor response after anti-CTLA-4 antibody therapy requires intact commensal microbiota.^270^ Anti-biotic treatment or the absence of living microorganisms compromised the anti-tumor efficacy of anti-CTLA-4 antibodies.^270^ Recolonization experiments with specific strains isolates showed that the introduction of certain commensal bacteria (e.g., *Bacteroides fragilis, Bacteroides thetaiotaomicron*, and *Burkholderia cepacia*) was able to recover anti-tumor responses.^270^ Another study reported that *Bifidobacterium* was linked to improved anti-tumor efficacy of anti-PD-L1 antibodies, and that orally administered *Bifidobacterium* improved the anti-tumor efficacy of ICIs by promoting the role of DCs and activating cytotoxic CD8^+^ T cells.^271^ These findings indicate that specific commensal bacteria may enhance the anti-tumor efficacy of ICIs.

Further research aimed to identify specific strains in human microbiota that can enhance the effectiveness of anti-tumor treatment. Research on patients with metastatic melanoma, has shown that certain bacterial strains (e.g., *Faecalibacterium prausnitzii* and *Gemmiger formicilis*) are linked to a favorable reaction to anti-tumor antibodies, while other strains (e.g., *Bacteroides*) are linked to adverse linked to ADRs.^272^ In addition, a greater α-diversity and the existence of particular bacterial (e.g., *Ruminococcaceae*) are also connected to a positive reaction to anti-PD-1 antibodies.^273^ In non-small cell lung cancer or urothelial tumor patients with clinically effective to ICIs, *Akkermansia muciniphila* (*A. muciniphila*) and *E. hirae* were found to be more abundant than normal.^274^ Despite the fact that *Bifidobacterium longum, Collinsella aerofaciens*, and *E. faecium* were enriched in melanoma patients who were responsive to anti-PD-1 antibodies, *Ruminococcus obeum* and *Roseburia intestinalis* were enriched in non-responders.^275^ The transfer of microbiota from tumor patients responsive to ICIs to GF- or antibiotic-pretreated mice resulted in enhanced anti-tumor efficacy.^273–275^ In addition, co-administration of a mixture of 11 strains acquired by healthy volunteers was found to enhance the anti-tumor effects of ICIs in GF mice by vigorously inducing the production of IFN-γ by CD8^+^ T cells.^276^ Although B cells have been considered to play a potential role in ICIs therapy, the relationship between B cells responses and gut microbiota has not been extensively studied.^277^

Collectively, these studies indicate that the mouse and human gut microbiota may play a crucial role in regulating tumor IVDR on ICIs by modulating the host immune system. However, there is limited coherence in the key microbiota subgroups reported in these studies, which may be due to factors such as geographical and population differences, specific microbiota associations with certain tumor types, and other aspects of the microbiota that are currently understudied.^278,279^ To further explore these associations, larger clinical studies and deeper sequencing analyses may be necessary.^280^

Although targeting gut microbiota to enhance the effectiveness of ICIs efficacy is a relatively new strategy, recent progress has been made. Two early clinical studies using fecal microbiota transplant (FMT) showed the potential for improved responses to ICIs therapy.^281,282^ In one trial, 10 individuals with anti-PD-1-resistant metastatic melanoma were administered FMT from a donor who had previously achieved complete remission with anti-PD-1 therapy.^281^ This trial revealed that FMT, along with anti-PD-1 reinduction therapy, is both secure and viable for certain patients, leading to heightened immune activity within the tumor. Different research involved the use of FMT derived from melanoma patients who had shown a long-term response to treat patients with anti-PD-1-resistant melanoma.^282^ Among the 15 patients, 3 achieved objective responses and 3 had long-lasting stable disease. These findings indicate that manipulating gut microbiota could potentially alter the tumor microenvironment and counteract resistance to ICIs treatment. However, larger patient cohorts are needed to identify the ideal composition of FMT and the microbiota profiles of patients that will improve ICIs efficacy.

### Pharmacomicrobiomics in cardiovascular system

Cardiovascular diseases (CVDs) are responsible for 31% of global mortality, causing over 17.7 million deaths worldwide.^283–286^ Studies show that alterations in gut microbiota are linked to an increased risk of CVDs, e.g., hypertension, atherosclerosis (AS), and heart failure by affecting IVDR and inflammation.^287–291^ Pharmacomicrobiomics holds promise for understanding how microbiota affects CVDs and how drugs can be developed to better target microbiota.^292^ However, more work is necessary to truly unlock the intricate interactions between microbiota, drugs, and CVDs. Next, we describe the mechanisms of drug-microbiota interactions for patients with CVDs (Table 3).

**Table 3:** List of example drug-microbiota interactions with well-characterized mechanisms in eight human body systems

Diseases	Drugs	Involved bacteria	Mechanisms of interaction with microbiota	PMID
Cardiovascular system diseases	Warfarin	*Bacteroides, Escherichia Shigella*, and *Klebsiella*	Weak response to the drug.	32505835
		*Enterococcus*	High response to the drug.	32505835
	Statins	*Bacteroides, Butyricimonas*, and *Mucispirillum*	Anti-inflammatory and improves hyperglycemia.	31551944
	Digoxin	*Eggerthella lenta*	Reduction of digoxin to its inactive metabolite and *E. lenta* is transcriptionally activated by digoxin.	23869020 6836275 32815419 10555354
	Captopril	*Parabacteroides, Mucispirillum*, and *Allobaculum*	Increased bacteria.	30755073
		*Clostridiales (Dehalobacterium, Oscillospira, Roseburia*, and *Coprococcus)*	Increased bacteria.	26301638
	Benazepril & Enalapril	Gut dysbiosis	Enalapril has been shown to reduce blood trimethylamine N-oxide (TMAO) levels.	29290360 29236735
Endocrine system diseases	Metformin	*Akkermentia muciniphila*	Contribute to the glucose-lowering effects of metformin.	28530702
		*Bacteroides fragilis*	*B. fragilis*-glycoursodeoxycholic acid (GUDCA)-intestinal farnesoid X receptor (FXR) axis improves anti-hyperglycemic effect.	30397356
	Acarbose	*Prevotella, Lactobacillus*, and *Faecalibacterium*	Acarbose increases SCFAs-producing bacteria.	10198028
		*Dialister*	Increased bacteria, had a negative correlation with Hemoglobin A1c (HbA1c).	28130771
		*Bifidobacterium* and *Lactobacillus*	Increased bacteria.	25327485 7256727
		*Bacteroidaceae, Enterobacteriaceae*, and *lecithinase positive Clostridium*	Decreased bacteria.	25327485 7256727
	Miglitol	*Erysipelotrichaceae* and *Coriobacteriaceae*	Decreasing harmful bacteria.	28349245
		SCFAs-producing bacteria	Increased bacteria.	28349245
	Liraglutide	SCFAs-producing bacteria, including *Bacteroides*, *Lachnospiraceae*, and *Bifidobacterium*	Changed the composition of gut microbiota, increased the *Bacteroidetes-*to*-Firmicutes* ratio.	27633081 29171288
	Sitagliptin	*Bacteroidetes*	Decreased bacteria.	30922964
		*Firmicutes* and *Tenericutes*	Increased bacteria.	30922964
		SCFAs-producing bacteria	Sitagliptin partially corrected the dysbiosis of microbiota.	27631013
	Vildagliptin	*Lactobacilli spp*.	Increased bacteria.	30186247
		*Oscillibacter spp*.	Decreased bacteria.	30186247
		*Bacteroidetes* and *Firmicutes*	Decreased *Firmicutes-to-Bacteroidetes* ratio.	29036231
Digestive system diseases	Sulfasalazine (SAS)	*Lactobacillus acidophilus L10, Bifidobacterium lactis B94*, and *Streptococcus salivarius K12*	Probiotics possessed azoreductase activity to metabolize sulfasalazine.	6129936
	Proton pump inhibitors (PPIs)	*Enterobacteriaceae, Enterococcaceae*, and *Lactobacillaceae; Rothia dentocariosa* and *Rothia mucilaginosa*, the genus *Actinomyces* and the family *Micrococcaceae*	Increased bacteria.	26719299 26657899 26164495
		*Ruminococcaceae* and *Bifidobacteriaceae*	Decreased bacteria.	
		*Clostridium difficile, Campylobacter*, and *Salmonella*	Increase susceptibility to enteric infections.	25337874 24096337
	Nonsteroidal anti-inflammatory drugs (NSAIDs)	*Clostridium perfrengens* and *Escherichia coli*	Bacteria in the intestine produce β-glucuronidases to de-glucuronidation of drugs leading to toxic metabolites.	30401818
Nervous system diseases	Levodopa, carbidopa, and entacapone	*Enterococcus faecalis* and *Lactobacillus*	Bacterial contain tyrosine decarboxylases (tyrDCs) that can convert levodopa to dopamine.	30659181 31196984
		*Eggerthella lenta*	Convert dopamine to m-tyramine, which can cause hypertensive crisis.	31196984
		*Ruminococcus torques*	Metabolize entacapone 84% efficiency.	31158845 29555994
Locomotor system diseases	Methotrexate (MTX)	*Enterobacterial* group*, Ruminococcaceae, Bacteroidetes* phyla, and *Bacteroides fragilis*	Decreased bacteria.	29351480 7601013 30049384
		*Lachnospiraceae* family	Increased bacteria.	29351480
		*Prevotella maculosa*	Enhancement of the response to the treatment.	26214836
Genitourinary system diseases	Abiraterone acetate (ABI), enzalutamide (ENZ), and degarelix acetate (DEG)	*Akkermansia muciniphila* and *Lachnospiraceae*	Depleted in patients treated with abiraterone acetate.	32973149
		*Ruminococcaceae* and *Akkermansia muciniphila*	More abundant in the microbiota of men taking oral ATT.	29089606 34618567
Respiratory system diseases	Isoniazid (INH), rifampin (RIF), and pyrazinamide (PZA)	*Clostridiales* and *Lactobacillus*	Decreased bacteria.	28683818 29555994
		*Porphyromonadaceae, Bacteroidetes, Proteobacteria, Gordonibacter*, and *Marvinbryantia*	Increased bacteria.	28683818 30910543
		*Bacteroides*	Produce polysaccharides that increase Treg cell responses and mediate mucosal tolerance.	22999859
		*Prevotella*	Increased Th17 cells-mediated inflammation.	28542929 21995674

#### Warfarin

Warfarin, a vitamin K antagonist, has become the mainline of oral anti-coagulant treatment for preventing and treating thromboembolic complications in 1954.^293–295^ However, its narrow therapeutic window and apparent IVDR prompted many studies to identify genetic factors that influence treatment outcomes.^296–298^ Through these studies, mutations predictive of therapeutic response have been identified in VKORC1 and CYP2C9, which encode metabolic enzymes associated with vitamin K and coumarin vitamin K antagonists, respectively.^299,300^ Nonetheless, approximately 35% of individuals with a delay in responding to warfarin have not been explained by these genetic factors.^301^ Recently, researchers have investigated the possible impact of gut microbiota response to warfarin, given the known association between gut microbiota and vitamin K metabolism. A trial of 200 patients with varying degrees of reaction to warfarin showed that *Bacteroides*, *Escherichia Shigella*, and *Klebsiella* were prominent in the lower-responders. *Escherichia Shigella* has the necessary enzymes to produce vitamin K, which may account for the weak response. In contrast, *Enterococcus* was connected to an increased reaction to warfarin.^302^ This research is the initial one to establish a connection between gut microbiota and IVDR to warfarin, a medicinal compound that has a limited therapeutic window and can result in bleeding or inefficiency if not dosed appropriately. Although these findings show promise, additional research is required to confirm and refine the results. Determining the impact of gut microbiota in warfarin response has the potential to improve individualized dosing strategies and optimize treatment outcomes.

#### Statins

Statins are commonly utilized for the first and second-line management of CVDs by reduction of low-density lipoprotein cholesterol (LDL-C), the so-called ‘bad’ cholesterol.^303–305^ By inhibiting 3-hydroxy-3-methylglutaryl coenzyme A (HMG-CoA), statins mainly reduce the de novo synthesis of cholesterol in the liver.^306^ However, studies in recent decades have shown that statins have a number of pleiotropic effects, including anti-inflammation, anti-thrombosis, and anti-oxidation, which may be involved in their cardiovascular protective properties.^307,308^ Statins have been proven to inhibit the function of Rho kinase and Rac1 protein, which are associated with the progression of AS, a major risk factor for CVDs. Statins also activate peroxisome proliferator-activated receptor-gamma (PPAR-γ), a nuclear receptor that contributes to the regulation of inflammation, lipometabolism, and glycometabolism.^308^ However, the mechanisms underlying such effects of statins are poorly understood.

Furthermore, recent research indicates that statins can modify the structure of gut microbiota, leading to changes in the proportion of certain species. One study found that statin treatment dramatically boosted the amount of certain anti-inflammation-related species (e.g., *Bacteroides*, *Butyricimonas*, and *Mucispirillum*), which may help to ameliorate hyperglycemia.^309^ Meanwhile, another research suggests that statins could cause gut dysregulation by altering the complexity of the BAs pool via a pregnancy X receptor (PXR) based mechanism,^310^ potentially leading to adverse health effects.^311^ BAs are synthesized from cholesterol in the liver and are important in the digestion and absorption of dietary fats. Direct inhibition of de novo cholesterol formation by statins could inhibit BAs synthesis in the liver, thereby altering the BAs pool in the human gut. Alterations in the number and structure of BAs in the gut may directly or indirectly induce considerable and complex physiological reactions by altering gut microbiota.

Despite growing evidence that statins might be beneficial for gut microbiota, more studies are warranted to investigate the long-term effects of statin treatment on gut microbiota and whether these changes are consistently beneficial for CVDs. In addition, it is critical to evaluate the possible risks and benefits of statin treatment and IVDR when making treatment decisions for the prevention and management of CVDs.

#### Digoxin

Digoxin, also known as digitalis, is a drug that can enhance the pumping efficiency of damaged or weak hearts, leading to better clinical stability and exercise capacity.^312,313^ However, in lower-flow congestive heart failure, digoxin may be ineffective in 10% of patients, due to deactivation by specific strains, highlighting the significance of IVDR from humans and microbiota. The deactivation of digoxin is mediated by the transformation of digoxin to an inactivated state, dihydro-digoxin, by *Eggerthella lenta* (*E. lenta*).^314–317^ In another groundbreaking research, an operon coding for the *E. lenta* cytochrome, cardiac glycoside reductase (cgr), was identified to be transcriptionally activated by digoxin, suggesting a predictable microbiota biomarker for digoxin deactivation. The researchers also explored the potential that endogenous digoxin-related substances might have been chosen for inactivation and determined if sensible dietary interference could regulate the inactivation of digoxin in vivo. As previously understood, *E. lenta* proliferation relies on arginine, which not only improves proliferation but also prevents digoxin deactivation. Hence, increased arginine concentrations, either dietary or microbiota-derived, may be able to suppress this unwanted microbiota function.^318,319^ To test this hypothesis, the researchers performed an in vivo experiment using mice given two distinct diets: one devoid of any protein and the other containing 20% of total calorie intake from protein. The results showed that an increase in protein intake notably raised levels of digoxin in the blood and urine, but only in mice colonized with digoxin-reducing strains.^318^ Overall, this research offers a new understanding of how *E. lenta* deactivates digoxin and emphasizes the possible utilization of dietary interference to regulate this microbiota process. These findings could have significant clinical applications for individuals with low-flow congestive heart failure who may benefit from digoxin therapy. Furthermore, the identification of predictive microbiota biomarkers for drug inactivation could lead to the identification of personalized treatment guidelines on the basis of individual microbiota.

#### Angiotensin-converting enzyme inhibitors (ACE-Is)

Captopril is a first-generation ACE-Is drug widely used to treat hypertension through the inhibition of the renin-angiotensin system (RAS) in centrally and peripherally sites.^320,321^ In addition to its anti-hypertensive effects, captopril has also been found to influence the composition of gut microbiota.^322–325^ Research has indicated that captopril maintains its anti-hypertensive activity after withdrawal. An animal experiment treating rats with captopril showed an increased abundance of *Parabacteroides, Mucispirillum*, and *Allobaculum*.^326^ In addition, captopril reduces neuronal inflammation in the autoregional area and diminishes sympathetic nerve drive, which balances the gut microbiota.^327,328^ Recent findings also suggest that mothers treated with captopril have a persistent anti-hypertensive response by reconstituting gut microbiota, improving gut pathologies and permeabilities, and restoring the dysregulated gut-brain axis in male offspring.^329^ For example, the presence of *Clostridia* and *Clostridiales* were higher in rats of maternal captopril. Pregnant rats treated with captopril exhibited a greater mean abundance of the order *Clostridiales* (e.g., *Dehalobacterium, Oscillospira, Roseburia*, and *Coprococcus*) in gut microbiota, in comparison to pregnant rats. As a result, captopril could affect gut microbiota growth and composition in humans, thereby altering the effectiveness of drugs.^330^ The effect of captopril on gut microbiota may be significant in managing hypertension and associated diseases.

Benazepril and Enalapril are second-generation of ACE-Is and are primarily for treating CVDs (e.g., arterial hypertension and congestive heart failure).^331^ These drugs work by inhibiting the activity of the angiotensin-converting enzyme (ACE), resulting in a lower generation of angiotensin II, a hormone that narrows the vessels and raises pressure.^332,333^ Recently, researchers have shown that both benazepril and enalapril have the capacity to positively influence gut microbiota.^334–337^ In rats, benazepril treatment was found to promote the restoration of gut microbiota structure by changing the balance of gut microbiota.^338,339^ Specifically, benazepril is primarily metabolized in the liver and transformed into diacid benazeprilat, which can affect gut microbiota. In contrast, enalapril has been shown to reduce blood levels of trimethylamine N-oxide (TMAO), a compound produced by the gut microbiota metabolizing certain nutrition.^340,341^ High concentrations of TMAO are linked to a higher incidence of CVDs. Enalapril influences plasma TMAO levels by modifying the composition of gut microbiota and controlling the excretion of methylamines in urine.^337^ These findings suggest that ACE-Is might be effective in improving gut microbiota composition and cardiovascular health. Therefore, by promoting the restoration of gut microbiota structure and reducing TMAO levels, benazepril and enalapril may help reduce the risk of CVDs.

### Pharmacomicrobiomics in endocrine system

The endocrine system is a complicated system of glands that produce hormones, which are responsible for regulating various bodily functions such as metabolism, growth, and development.^342–344^ Current evidence suggests that microbiota plays an important role in the regulation of the endocrine system and can influence the IVDR of endocrine disorders.^345^ The study of pharmacomicrobiomics in the endocrine system has the potential to enhance our knowledge of the underlying mechanisms of IVDR and to advance precision medicine based on the characteristics of microbiota. Further research in this field is necessary to fully realize the potential benefits of this approach. Below, we will list several cases of interaction between anti-diabetics and gut microbiota (Table 3).

#### Metformin

Metformin (1,1-Metformin Hydrochloride), a widespread anti-diabetic drug, is also known to have other benefits, such as reducing obesity, and reducing the incidence of CVDs and cancers.^346,347^ However, the precise mechanisms by which metformin works to regulate glucose levels are not yet fully understood.^347–351^ While some theories suggest that metformin activates AMP-activated protein kinase (AMPK) and inhibits the mitochondrial respiratory chain, these completely account for all of its positive actions.^352^ Thus, researchers have investigated mechanisms underlying gut microbiota regulation of glucose metabolism and energy balance, particularly the potential involvement in IVDR.^353–355^ Recently, some work has highlighted how gut microbiota might influence the anti-hyperglycemic effect of metformin. For example, Wu et al. transplanted feces from untreated Type 2 Diabetes Mellitus (T2D) patients to GF mice and observed significant improvement in glucose tolerance compared to control mice. They identified the expression of *A. muciniphila* and its metal-binding proteins as key factors in metformin-microbiota interactions. This contributed to explaining earlier unresolved questions about the relationship between metformin effectiveness and metal balance.^353^ Another study by Sun et al. showed that the anti-hyperglycemic effect of metformin involves a new mechanism named the *Bacteroides fragilis*-glycoursodeoxycholic acid (GUDCA)-intestinal farnesoid X receptor (FXR) axis. This pathway relies on gut microbiota and involves the activation of intestinal FXR signaling to ameliorate metabolic dysfunction. Metformin was found to reduce *Bacteroides fragilis*, preventing GUDCA degradation.^354^

In addition to its anti-hyperglycemic effect, metformin demonstrated protective efficacy against CVDs through anti-inflammatory mechanisms including activating the AMPK pathway and inhibiting nuclear factor-κB (NF-κB) pathway.^356–358^ Growing evidence indicates that microbiota-mediated pathways might be implicated in the therapeutic effects of metformin. For example, changes in gut microbiota structure due to metformin treatment may lead to alterations in glucose, hormones, BAs pool, SCFAs, and immunity.^351,352,359^ Collectively, emerging evidence suggests that the gut plays a vital role in metformin therapy and that microbiota-mediated mechanisms involving changes in gut microbiota structure may be responsible for some of its pleiotropic effects. This has important implications for developing new therapies that target gut microbiota and their interaction with IVDRs.

#### α-Glucosidase inhibitors (AGIs)

AGIs are a class of drugs that alter the absorption and metabolism of carbohydrates in the small intestine.^360,361^ These drugs have been found to significantly affect the structure and variability of gut microbiota, which may have important implications for managing metabolism diseases (e.g., T2D). Acarbose and miglitol are two commonly prescribed AGIs that have been widely evaluated for their impact on gut microbiota. In vitro, experiments have shown that acarbose selectively inhibits the development of *E. coli* by inhibiting the maltose importer, while clinical trials have indicated that acarbose enhances the abundance of microbiota that produce SCFAs, e.g., *Prevotella, Lactobacillus*, and *Faecalibacterium*.^362,363^ SCFAs are known to be beneficial for metabolic health, and treatment with *lactobacillus* has been shown to reduce glycemia.^364–366^ Furthermore, a study demonstrated that *Dialister*, a taxon found in gut microbiota, was enhanced after acarbose therapy and had a negative correlation with Hemoglobin A1c (HbA1c), suggesting a possible role in controlling glucose metabolism.^362^ Acarbose has also been shown to increase *Bifidobacterium* and *Lactobacillus*, and reduce harmful *Bacteroidaceae, Enterobacteriaceae*, and *lecithinase positive Clostridium* in stool collected from people with hyperlipidemia or T2D.^367,368^

Miglitol, on the other hand, has anti-inflammatory properties in mice, inhibiting histological and molecular indicators of inflammatory reaction and decreasing intestinal transit time generated by high-fat and high-glucose diet (HFHSD).^369^ Consuming an energy-dense diet has been shown to increase *Erysipelotrichaceae* and *Coriobacteriaceae*, but miglitol has been found to reverse this effect. It has been hypothesized that the reduction in intestinal inflammation is connected to these alterations in gut microbiota. However, the effect of miglitol on the heterogeneity and structure of gut microbiota remains unknown and requires further study. Overall, the results of these findings indicate that AGIs, particularly acarbose, may be useful in reestablishing gut microbiota balance in individuals with T2D. By increasing SCFAs-producing microbiota and decreasing potentially harmful microbiota, these drugs can potentially impact metabolic health with positive effects. More studies are necessary to fully elucidate the IVDR of AGIs on gut microbiota and their impact on metabolic health.

#### Glucagon-like peptide-1 receptor agonists (GLP1-RAs)

Glucagon-like peptide-1 (GLP-1) is an incretin hormone produced by enteroendocrine cells as a result of dietary.^370–372^ Its role in enhancing glucose-induced insulin secretion, inhibiting glucagon secretion, and suppressing appetite and gastric emptying makes it a powerful target in treating diabetes and overweight.^370,373^ Recent studies have emphasized the function of gut microbiota in regulating satiety and glucose homeostasis by inducing GLP-1 secretion.^374–377^ Furthermore, GLP1-RAs (e.g., liraglutide), an emerging sub-class of anti-diabetic drugs, have been shown to modulate the intestinal microenvironment and alter gut microbiota composition.^378–382^ In general, the *Firmicutes*-to-*Bacteroidetes* ratio is considered an important indicator of gut microbiota structure. Studies have shown that liraglutide can modify gut microbiota to a leaner proportion in normally-weighed diabetes mice, resulting in a higher *Firmicutes*-to-*Bacteroides* ratio.^378^ Interestingly, another research demonstrated that liraglutide increased the *Bacteroides*-to-*Firmicutes* ratio to decrease body weight in both simply overweight and diabetes-overweight individuals independent of glycaemic condition.^380^ Such differences are probably attributed to varying levels of hyperglycemia and different model systems used. GLP1-RAs have been proven to profoundly change the structure of gut microbiota in diabetic male rats.^379,383^ These changes include selective enhancement of several SCFAs-producing microbiota, e.g., *Bacteroides*, *Lachnospiraceae*, and *Bifidobacterium*.^379^ Furthermore, GLP1-RAs can re-establish, at least in part, the homeostasis of gut microbiota.^383^

#### Dipeptidyl peptidase-4 inhibitors (DPP4Is)

DPP4Is (e.g., sitagliptin and vildagliptin) are first-line hypoglycaemic drugs approved by the American Association of Clinical Endocrinologists for T2D.^370,384^ DPP4Is decrease blood glucose primarily via inhibition of GLP-1 degradation.^385^ Previous studies have suggested the DPP-4-related function of gut microbiota may be a goal for DPP4Is, potentially opening up novel therapeutic applications for DPP4Is to modulate gut microbiota discordance. Studies have shown that DPP4Is can improve glycaemic control through increased *Bacteroidetes*, thereby greatly reverting high-fat diet (HFD)-induced alterations in gut microbiota.^386^ The study of the effect of sitagliptin on gut microbiota showed decreased *Bacteroidetes*, and increased *Firmicutes* and *Tenericutes*. However, sitagliptin moderately restored microbiota imbalance and modified the abundance of SCFAs-producing microbiota in T2D rats.^387^ Similarly, vildagliptin administration was related to increased *Bacteroidetes* and decreased *Firmicutes* coupled with decreased *Firmicutes-to-Bacteroidetes* ratio in diabetic rats.^388^ Moreover, vildagliptin has been reported to produce benefits by modulating gut microbiota, leading to increased *Lactobacilli* and decreased *Oscillibacter*.^385^ To clarify the mechanisms of variation, researchers experimentally confirmed that vildagliptin inhibited Toll-like receptor (TLR) ligands in caecal content and restoration of antimicrobial peptide levels and ileum crypt depth.^385^ Studies have also shown that DPP4Is may indirectly reduce the secretion of pro-inflammatory cytokines in the liver through their effect on gut microbiota. Overall, these studies highlight the importance of DPP4Is on gut microbiota and reveal promising strategies to improve glucose homeostasis and IVDR.

### Pharmacomicrobiomics in digestive system

The digestive system is one of the most extensive areas of pharmacomicrobiomics because it is home to complicated and varied microbiota that is critical in drug metabolism and efficacy.^189,389^ Gut microbiota has also been demonstrated to impact outcomes of drugs used to treat a variety of digestive diseases, e.g., inflammatory bowel diseases (IBD) and irritable bowel syndrome (IBS).^390^ Patients with IBD have been detected to have altered gut microbiota composition and function, which may affect IVDR. This has contributed to the advancement of microbiota-based treatments for IBD, e.g., FMT, which involves transplanting a healthy gut microbiota into a patient to restore microbial balance and improve treatment outcomes. Overall, the field of pharmacomicrobiomics in the digestive system has enormous potential for improving drug efficacy and reducing ADRs. However, deeper studies are necessary to better comprehend the complicated between gut microbiota and drugs, as well as to develop microbiota-based therapies for various digestive and metabolic disorders (Table 3).

#### Sulfasalazine (SAS)

SAS is a medication that was initially developed with the aim of treating inflammatory conditions caused by bacterial infections. However, its effectiveness was later discovered in treating ulcerative colitis (UC).^391^ The drug is composed of aminosalicylate and sulfapyridine, which are linked together by an azo bond. One of the unique properties of SAS is that it is not rapidly absorbed in the up-stream gastrointestinal tract. Instead, it is broken down into its components by gut microbiota in the colon. Sulfapyridine is then absorbed into the bloodstream, while mesalazine (5-aminosalicylic acid, 5-ASA) can be inactivated in the colon.^392^ A PK study in health subjects revealed that gut microbiota are critical in activating SAS. This discovery might explain why this drug is better at treating UC than Crohn’s disease (CD).^393^ The ability of gut microbiota SAS could be enhanced by administering probiotic strains (e.g., *Lactobacillus acidophilus L10, Bifidobacterium lactis B94*, and *Streptococcus salivarius K12*). In an in vitro trial, researchers incubated the contents of a rat colon with SAS alone or SAS combined with probiotics under anaerobic conditions.^394^ They found that the probiotics possessed azoreductase activity, allowing them to metabolize SAS. The samples incubated with SAS plus probiotics had a greater level of 5-ASA and sulfapyridine restored. Despite SAS being an efficient and inexpensive therapy for UC, some patients have reported ADRs when using it, such as nausea, skin rash, and anorexia.^395^ This may be caused by the metabolism of SAS through gut microbiota. As a result, SAS is less popular than other treatments for UC.

#### Proton-pump inhibitors (PPIs)

PPIs are widespread drugs for the treatment of acid-induced diseases including peptic ulcers, gastro-oesophageal reflux, dyspepsia, gastro-duodenopathy, and bleeding.^396–398^ Due to their effectiveness and safety properties, PPIs therapy has expanded rapidly in the last decades. Controversies have arisen, particularly relating to the safety profile and possible ADRs of chronic therapy with PPIs. Up to 70% of PPIs prescriptions have been estimated to be unneeded.^399^ Once started, the original indication for PPIs is rarely re-evaluated, with later withdrawal attempts resulting in needless long-term use.^400,401^

A large-scale patient-based clinical trial in the Netherlands shown that PPIs are among the drugs in greatest correlation with reduced gut microbiota diversity and taxonomic changes.^402^ Studies analyzing *16* *S* data from various cohorts found that PPI use led to decreased abundance of gut microbiota and increased oral microbiota.^403–405^ For example, *Enterobacteriaceae, Enterococcaceae*, and *Lactobacillaceae* increased, *Ruminococcaceae* and *Bifidobacteriaceae* decreased, while alterations towards typically oral microbiota are represented by *Rothia dentocariosa*, *Rothia mucilaginosa*, *Actinomyces*, and *Micrococcaceae* increased.^404^ In addition, the observed changes appear to be a class-efficacy of PPIs, with increased doses linked to greater microbiota changes.^115^ Recent research has shown that PPIs use is highly relevant to 24 taxa and 133 pathways, with predictable function alterations including increased biosynthesis of fatty acid, lipid biosynthesis, and L-arginine, and degradation of purine de-oxyribonucleoside.^115^ Another in vitro experiment testing direct affected by popular drugs on gut microbiota demonstrated significant alterations in growing speeds, meaning that bonding of PPIs to bacterial H^+^/K^+^ ATPases may mediate the directly influence.^406^ The reduction in gastric acidity caused by PPIs allows oral microbiota to colonize gut microbiota, resulting in altered taxonomic homeostasis and potentially increased susceptibility to intestinal infections, e.g., *Clostridium difficile* (*C.* *difficile*), *Campylobacter*, and *Salmonella*.^407–410^ Moreover, PPIs initiation and withdrawal may cause alterations in gut microbiota, potentially exacerbating liver cirrhosis.^411^ Furthermore, long-term PPIs use, particularly in childhood, can cause permanent alterations in the growing gut microbiota, potentially leading to obesity later in life.^412^ Although PPIs are widely recognized as harmless and efficient, medical practitioners should reassess the extensive, long-term impact and OTC accessibility. The rapid and widespread shift to PPIs use has led to variations in changes in gut microbiota, affecting up to one-fifth of the population. Therefore, it is important to meticulously evaluate the long-term impact of PPIs-induced alterations on gut microbiota, especially during early development, and their potential effects on health and disease in later life.

#### Non-steroidal anti-inflammatory drugs (NSAIDs)

NSAIDs are generally used to alleviate pain and inflammation, but their use can cause ADRs, such as stomach ulcers and damage to the mucous membrane of the small intestine.^413,414^ This is due to the fact that the bacterial glucuronidase metabolizes NSAIDs in the intestine, similar to the case of CPT-11, which converts the drug into toxic metabolites that damage the intestinal mucosa.^415^ The glucuronides of NSAIDs produced in the liver reach the intestine via the bile, where bacterial GUSs hydrolyse them into aglycones.^416^ These aglycones are then reabsorbed and converted by cytochrome P450 into potentially cytotoxic intermediates that cause intestinal toxicity.^417^ Like CPT-11, a recent study demonstrated that Inh1 (Inhibitor 1), a novel microbe-specific GUSs inhibitor, can reduce the intestinal ADRs of diclofenac.^418^ This suggests that inhibiting GUSs is a promising approach to reducing the adverse effects of certain drugs. While gut microbiota produces GUSs with beneficial functions to the organism, opportunist or intestine-pathogenic species, e.g., *Clostridium perfrengens* and *E. coli*, are responsible for the de-glucuronidation of drugs producing harmful metabolites. This is due to intracellular variations of GUSs, including variations in conformation, hydrophobicity, and mobility.^419^ The enhancing potential of bacterial GUSs-induced ADRs, especially gut injury, is not unique to NSAIDs and CPT-11. Other drugs including Regorafenib, an anti-tumor tyrosine kinase inhibitor, and venotonic flavonoids, have also been shown to be substrates for GUSs.^420–422^ Such evidence emphasizes the value of understanding the importance of bacterial enzymes in IVDR to develop strategies to reduce ADRs.

### Pharmacomicrobiomics in nervous system

Although pharmacomicrobiomics research is still in its infancy, preliminary studies indicate that microbiota may play a key role in regulating IVDR in the nervous system. One example of this is the use of probiotics to enhance the effectiveness of anti-depressant medication.^423^ Several researches have indicated that certain strains of gut microbiota can produce neurotransmitters, e.g., serotonin and gamma-aminobutyric acid (GABA), which are known to regulate mood and anxiety. By supplementing these strains with probiotics, the researchers hope to increase the availability of these neurotransmitters and thereby improve the response to treatment with anti-depressants. Another area of interest is the effect of the microbiota on neurodegenerative diseases, e.g., Parkinson’s disease, for which the commonly used therapeutic drug is levodopa (Table 3).

#### Levodopa, carbidopa, and entacapone

Levodopa is a drug for the treatment of Parkinson’s disease, a neurological disorder described by tremors, stiffness and difficulty in moving.^424,425^ Levodopa is taken orally and must be reabsorbed through the small intestine to reach the brain, where it is transformed into dopamine by the tyrosine decarboxylases (tyrDCs). The effectiveness of the drug depends on its bioavailability to the brain, and the drug is usually used alongside inhibitors of catechol metabolic (e.g., carbidopa and entacapone) to inhibit extra-brain metabolism. Emerging evidence suggests that gut microbiota can metabolize levodopa. Certain species, e.g., *Enterococcus faecalis* (*E. faecalis*) and *Lactobacillus*, have been found to contain tyrDCs.^426,427^ However, this microbiota metabolism reduces the amount of levodopa that is available to the brain, resulting in decreased drug efficacy. Additionally, microbiota metabolism of levodopa can also lead to ADRs, as *E. lenta* and other ten species can further convert dopamine to m-tyramine, which can cause hypertensive crisis.^427^

It is interesting to note that gut microbiota can also directly metabolize carbidopa and entacapone. For example, *E. faecalis* has been shown to metabolize both levodopa and entacapone with 98.9% efficiency.^42^ In turn, entacapone would reduce some species, e.g., *Ruminococcus torques*, which can still metabolize entacapone with 84% efficient.^42,406^ Such findings illustrate the complex interactions between gut microbiota and IVDR, which can influence drug efficacy and safety. While the field of pharmacomicrobiomics in the nervous system is still in its early stages, it has the opportunity to broaden our insight into drug-microbiota interactions and lead to the emergence of novel therapeutic strategies for neurological and psychiatric disorders.

### Pharmacomicrobiomics in locomotor system

The locomotor system is an indispensable part of the human body, consisting of bones, muscles, and joints, which work together to allow movement.^428^ The health of the locomotor system can be affected by various diseases, e.g., osteoarthritis (OA), rheumatoid arthritis (RA), and osteoporosis, which can result in pain, stiffness, and reduced mobility. In recent years, a growing focus has been placed on the impact of gut microbiota in the pathology and treatment of these diseases.^429^ One example of such a drug is methotrexate (MTX), which is commonly used to treat RA (Table 3). Evidence indicates that gut microbiota can influence the effect of MTX, the details of which will be described later.

Another example is NSAIDs, which are frequently applied to treat pain and inflammation related to various locomotor system disorders. However, these drugs can also cause gastrointestinal side effects, such as ulcers and bleeding. Research indicates that gut microbiota plays a crucial role in the metabolism of NSAIDs and can affect their efficacy and safety. Similar to the description of NSAIDs in digestive system diseases above, this will not be repeated.

#### Methotrexate (MTX)

MTX is a potent cytotoxic drug used to treat autoimmune diseases (e.g., RA).^430^ Its underlying action is based on inhibiting dihydrofolate reductase and thymidylate synthetase, thereby preventing de novo synthesis of pyrimidines and purines. While MTX is effective, it also has a high incidence of ADRs, including gastrointestinal, hematological, nephrotoxicity, and hepatotoxicity.^431^ One potential factor that may impact the response to MTX is gut microbiota. Research has indicated that MTX treatment could modify the composition of gut microbiota, decreasing the abundance of *enterobacterial*, especially *E. faecium*, and increasing the abundance of *Lachnospiraceae*.^432^ However, the decrease in beneficial strains (e.g., *Ruminococcaceae, Bacteroidetes*, and *Bacteroides fragilis*) may accentuate the gastrointestinal ADRs, particularly intestinal mucositis.^433,434^

Interestingly, the association between MTX and gut microbiota is bidirectional.^435,436^ The variability of gut microbiota may affect MTX therapy responses. The study found that patients with greater gut microbiota diversity, who had statistically abundant *Prevotella maculosa*, responded better to MTX.^436^ Despite the promising results, the response rate to MTX has highly IVDR, between 10% and 80%, with just 40% of patients obtaining a therapy blood concentration, and the ADRs are significant.^60,437^ Genetic factors have been explored to predict the IVDR to MTX, but they have not achieved clinical relevance.^438–440^ More investigations are necessary to assess the feasibility of using gut microbiota as a biomarker for MTX response and to determine its clinical significance. Nevertheless, promising evidence indicates that gut microbiota may play an important role in the PD of MTX, and further exploration may contribute to the emergence of personalized therapy for patients.

### Pharmacomicrobiomics in genitourinary system

The genitourinary system is an essential system for reproduction and waste excretion.^441^ Recently, evidence indicates that microbiota of the genitourinary system affects the efficacy and toxicity of several drugs, leading to the emergence of a field of pharmacomicrobiomics in this system. In particular, genitourinary microbiota has been shown to affect the effectiveness of androgen deprivation therapy (ADT), a frequent therapy for prostate cancer. ADT typically involves the use of androgen synthetic inhibitor (e.g., abiraterone acetate) or androgen receptor antagonist (e.g., enzalutamide, degarelix acetate) (Table 3).

#### Abiraterone acetate (ABI), enzalutamide (ENZ), and degarelix acetate (DEG)

Recently research has revealed the influence of ADT on the immune system and gut microbiota, and how this affects the progression of prostate cancer (PCa).^442^ ABI is a widely used drug in ADT, and research by Terrisse et al. underlined the crucial role of thymus-dependent T cells in regulating PCa progression, as CD4^+^ and CD8^+^ T cells consumption leads to a partial decrease in tumor development controlled by IVDR.^443^ Furthermore, the beneficial gut microbiota, e.g., *A. muciniphila* and *Lachnospiraceae*, were depleted in patients treated with ABI. However, this depletion could be reversed by the effects of the drug, which remodels the gut microbiota and promotes the proliferation of anti-inflammatory *A. muciniphila*, thereby elevating microbiota production of vitamin K2.^444^ Furthermore, the research suggested that specific gut microbiota, such as those with higher α-diversity and *A. muciniphila*, were related to better efficacy.^444,445^ Further studies by Sfanos et al. found that the genitourinary microbiota composition of men taking oral androgen-targeted therapy (ATT) differed from those taking Gonadotropin-releasing hormone (GnRH) agonists or antagonists monotherapy or not taking ADT. Species able to synthesize steroids, e.g., *A. muciniphila* and *Ruminoccocaceae*, were enriched in microbiota of men treated with orally ATT, which could affect disease progression and immunotherapies.^445^

Another study showed that gut microbiota could regulate the levels of circulated sexual hormones via effects on human cells, but also by direct biotransformation or synthesis, thereby promoting ADT resistances.^446^ PCa patients demonstrated higher levels of strains e.g., *A. muciniphila* and *Ruminoccocaceae*, which are capable of synthesizing steroid hormones via CYP17A1-like bacterial enzymes. Treating cultivated bacteria with CYP17A1 inhibitor ABI inhibited androgen biosynthesis. However, the response of the microbiota to reduced androgen levels leading to the expansion of strains capable of synthesizing androgens, has yet to be elucidated. In conclusion, the ability of the microbiota to regulate circulating sex hormones and the expansion of bacteria capable of synthesizing androgens can drive resistance to ADT. Future studies are warranted to clarify the intricate interactions between gut microbiota, PCa, and therapy.

### Pharmacomicrobiomics in respiratory system

In the respiratory system, microbiota plays an important role in maintaining health by modulating immunity, protecting against pathogens, and maintaining the mucosal barrier.^447–449^ Studies have shown that drugs may influence the composition and function of respiratory microbiota, and conversely, microbiota may impact drug metabolism and response. For example, anti-biotics can reduce microbiota diversity and enhance the growth of opportunistic pathogens that can cause chronic respiratory diseases. Inhaled corticosteroids have been shown to increase microbiota diversity and decrease the abundance of pathogenic bacteria, which may contribute to their therapeutic efficacy. Furthermore, the respiratory microbiota can impact drug delivery to the lungs. One of the best-known examples is anti-tuberculosis (anti-TB) therapy with the anti-biotic isoniazid (INH), rifampin, and pyrazinamide (PZA) (Table 3).

#### Isoniazid (INH), rifampin (RIF), and pyrazinamide (PZA)

The impact of anti-TB anti-biotics on the structure of gut microbiota has been studied in mice infected by *Mycobacterium tuberculosis*.^450,451^ The study found that treatment with INH, RIF, and PZA for three months led to significant and persistent eco-dysbiosis in a mouse model. The composition of gut microbiota was altered, with decreased *Clostridiales* and *Lactobacillus*, while increasing *Porphyromonadaceae, Bacteroidetes*, and *Proteobacteria*.^452^ The study further showed that a single dose of RIF decreased microbiota diversity, while either INH or PZA alone resulted in alterations to microbiota composition compared to gut microbiota of mice not treated with anti-biotics. Specifically, INH was found to enrich *Gordonibacter*, while PZA was found to enrich *Marvinbryantia*.^406,452,453^ Although TB infection did not dramatically alter gut microbiota composition, it was found to modulate the mucosal immune response. In humans, first-line anti-TB pharmacotherapy seems to have a minimal impact on gut microbiota.^454^ However, some strains have been demonstrated to regulate immunological responses in the host, such as *Bacteroides*, which produce polysaccharides that increase Treg cell responses and mediate mucosal tolerance.^455^ *Prevotella* has been associated with enhanced Th17 cells-mediated inflammation, while *Lactobacillus* promotes innate and adaptive immunological responses.^456,457^

Studies have also found that anti-TB drugs can reduce the microbiota necessary for intestinal homeostasis, increasing the risk of recurrent TB.^458^ T cells in previously treated patients also recognize type-2 epitopes poorly compared to patients without TB. The type 2 epitopes in the human gut are associated with *mycobacteria*. Multi-year trials of anti-TB treatment can contribute to a deeper understanding of the importance of microbiota in the progression of TB. A combination of anti-TB anti-biotics and host modifiers that target the host pathway may reduce treatment time and severity of TB, as well as the risk of reinfection. Emerging studies on host-pathogen correlations, host immunology, and host-targeted therapies indicate that this approach may be beneficial in the treatment of TB.^459^

## Mechanisms of action in pharmacomicrobiomics

Pharmacomicrobiomics is an emerging area that aims to comprehend the intricate relationships among drugs, microbiota, and host physiology.^460^ Understanding the multiple mechanisms of action in pharmacomicrobiomics is critical to the advancement of effective and safe treatment strategies that take into account the multiple mechanisms of interaction between IVDR and microbiota.

### Mechanisms of microbiota influence on IVDR

Gut microbiota is known to affect the effectiveness and acceptability of drugs by enzymatic modification structure and alteration of bioactivity or toxicity. A list of specific families of enzymes encoded by the microbiota and their effects on drugs is presented in Table 4. Conclusively, gut microbiota can impact IVDR in several ways, including (1) Alteration of drug metabolism: Microbiota can influence the activities of enzymes that metabolize drugs within the body, which can influence the efficacy and side effects of drugs.^461,462^ For instance, the microbiota can produce beta-lactamases that break down beta-lactam anti-biotics, rendering them ineffective.^463–466^ (2) Induction of drug resistance: Microbiota can develop resistance to drugs over time, making them less effective in treating infections.^282,467^ This resistance may be due to mutations in the DNA of microbiota or the acquisition of resistance genes from others. (3) Modification of drug targets: Microbiota can modify the drug targets in the body, making them less susceptible to the drug’s effects. For example, the microbiota can modify the target site of fluoroquinolone anti-biotics, thereby reducing their ability to bind and inhibit the enzyme.^468,469^ (4) Interaction with PK: Microbiota can also impact PK, which can affect drug efficacy and safety. For example, gut microbiota can modify the ADME of drugs, altering their therapeutic effects and toxicity.^470^ (5) Destruction of the host microbiota: The use of anti-biotics can interfere with the natural ecosystem of the host’s microbiota, leading to the overgrowth of pathogenic species (e.g., *C. difficile*).^471–473^ This can cause infections and other ADRs, which can impact drug safety. (6) Modulation of host immune response: Gut microbiota can modulate immune function in the host, which may influence the activity and toxicity of drugs that target the immune system.^474^ For example, gut microbiota can modulate the effectiveness of ICIs in tumor treatment by modulating the host immune response.^61,475–478^ (7) Regulation of gut barrier function: Gut microbiota may influence the integrity of the gut barrier, thereby affecting drug absorption and gut-host interactions.^479,480^ For example, gut microbiota can regulate the level of tight junction protein expression in the intestine, thereby affecting drug absorption.

**Table 4:** Some enzyme families encoded in gut microbiota and their impact on drugs

Microbiota enzyme	Effects on drugs
Beta-glucosidases	These enzymes hydrolyze sugar moiety that are often present in plant glucosides.
Beta-glucuronidases	These enzymes hydrolyze glucuronic acid, which are often formed during drug metabolism in the liver. Cleavage by beta-glucuronidases can increase drug reabsorption in the gut, leading to higher systemic exposure and potentially increasing the risk of toxicity.
Beta-lactamases	These enzymes break down beta-lactam anti-biotics, rendering them ineffective.
Sulfatases	These enzymes cleave sulfate groups of phase II host metabolism.
Azoreductases	These enzymes can reduce azo bonds that are present in some drugs.
Nitroreductases	These enzymes can reduce nitro groups that are present in some drugs, resulting in more toxic or inactive metabolites.
Proteases	These enzymes can cleave proteins and hydrolyze polypeptide chains.
Glycosidases	These enzymes can reduce the polarity of molecules.
C–S beta-lyases	These enzymes can cleave C-S bonds in drugs, generating ammonia for bacterial growth.
Cytidine deaminase	These enzymes catalyze conversion of cytidine to uridine by the of an amino group (-NH2) from the tidine molecule.
Tyrosine decarboxylases	These enzymes catalyze the conversion of the amino acid tyrosine to the biogenic amine tyramine by removing a carboxyl group (-COOH) from tyrosine.
Transferases	These enzymes can transfer functional groups, such as methyl or acetyl groups, to drugs, impacting their activity and toxicity.

In summary, microbiota can impact IVDR through a variety of complex and multifaceted mechanisms. A deeper insight into such relationships may help in the discovery of more beneficial and safer drugs, as well as in the management of IVDR.

### Mechanisms for drug-altered gut microbiota

There has been an increasing emphasis on the influence of drugs on gut microbiota and its influence on human healthcare. In a significant and comprehensive study, the authors evaluated the anti-microbial properties of more than 1000 drugs, among them 835 targeted agents that affect human cells.^406^ Of these, 24% showed anti-bacterial effects, influencing the proliferation of a minimum of 50% of bacterial strains tested. To sum up, several mechanisms are involved by which drugs affect gut microbiota, (1) Direct anti-biotic effects: Anti-biotics can selectively kill off certain microbiota, including both harmful and beneficial species.^392,481,482^ This can lead to imbalances in gut microbiota, which can have negative effects on human health. (2) Altered gut motility: The speed at which food passes through the intestine affects the proliferation and function of gut microbiota. Certain drugs, such as opioid painkillers, can slow down gut motility, which can lead to overgrowth of harmful bacteria.^483,484^ This may result in gastrointestinal symptoms like constipation and bloating, and in some cases can cause infections (e.g., *C. difficile*).^485^ (3) Modulation of immune function: Gut microbiota has a key function in modulating gut immunity. Several drugs, such as ICIs, can affect gut immunity which in turn can affect gut microbiota.^486^ For example, ICIs can decrease gut inflammation, which can lead to alterations in gut microbiota.^487^ (4) Changes in pH: The pH value in the intestine affects the growth and survival of different types of bacteria. Some drugs, such as PPIs used to treat acid reflux, can change the pH value of the gut, which affects the proliferation of different microbiota, thereby affecting the overall composition of gut microbiota.^404^ (5) Interference with microbial metabolism: Gut microbiota has a vital function in metabolizing compounds in the gut. Several drugs, such as NSAIDs, can interfere with microbial metabolism, which may have an effect on gut microbiota. In particular, NSAIDs can interfere with gut microbiota metabolism of BAs, leading to changes in gut microbial community.^488^ (6) Dietary changes: Certain drugs, such as laxatives or anti-diarrheal agents, can change the dietary environment in the gut.^489,490^ This may influence gut microbiota by changing the availability of nutrients and other compounds that gut microbiota use to grow and survive.

It’s important to note that a drug’s impact on gut microbiota can be either positive or negative. For example, certain probiotics or prebiotics are able to actively regulate gut microbiota, which may have a positive effect on the individual’s well-being.^491^ However, taking anti-biotics or other drugs that negatively affect gut microbiota can result in dysbiosis, which negatively affects human health. Therefore, it is essential to carefully evaluate the potential impact of drugs on gut microbiota and to develop personalized drug treatments that take into account an individual’s gut microbiota composition and function.

## Challenges and strategies

This review section identifies challenges and offers strategies for professionals, clinicians, and scientists involved in microbiota research and its influence on human well-being.

### Challenges

Pharmacomicrobiomics is a fast-developing discipline that aims to comprehend the intricate interaction between gut microbiota and drug response. While there have been significant advancements in this area, several challenges remain: (1) Lack of standardized methodologies: Currently, there is a lack of standardization in sample collection, sequencing, and data analysis in pharmacomicrobiomics studies. This creates a challenge in comparing findings among different studies and hinders the development of guidelines for clinical practice. (2) Complexity of gut microbiota: Gut microbiota is an intricate and ever-changing ecosystem that is impacted by various elements, including diet, lifestyle, and host genetics. It is difficult to disentangle the effects of these factors from drug-induced changes in the microbiota. (3) IVDR: There is significant inter-individual variability in gut microbiota, which can impact drug-microbiota interactions. (4) Limited understanding of mechanisms: Despite increasing evidence of drug-microbiota interactions, there is still a lack of comprehensive understanding regarding the mechanisms behind these interactions. It is also unclear how these interactions vary across different drugs and different patients. (5) Clinical translation: Although the potential clinical applications of pharmacomicrobiomics are promising, there are still many obstacles to be overcome before these findings can be translated into clinical practice. For example, the development of microbiota-based biomarkers for drug response will require large-scale validation studies and regulatory approval. (6) Ethical considerations: As with any field of personalized medicine, there are ethical considerations when using microbiota data to make clinical decisions. These include issues around privacy, data sharing, and potential discrimination based on microbiota characteristics. (7) Sample size: Numerous pharmacomicrobiomics studies often have limited sample sizes, potentially restricting their statistical power and the ability to generalize findings. Additional research of greater scale is required to confirm and expand upon preliminary results. (8) Diversity of populations: Most studies in pharmacomicrobiomics have been conducted on Western populations, and there is a lack of diversity in terms of ethnicity, geography, and lifestyle factors. This limits our understanding of how drug-microbiota interactions may vary across different populations. (9) Confounding factors: Various elements, such as diet, age, sex, and environmental exposures, can have an impact on gut microbiota and confuse the interpretation of the results, making it challenging to distinguish the effects of the drug from other factors.

Overall, addressing these challenges will require collaboration between researchers, clinicians, and industry partners to develop standardized methodologies, improve our understanding of the mechanisms underlying drug-microbiota interactions, and ensure that findings are translated into safe and effective clinical practice.

### Strategies

In light of these facts, to overcome these challenges, several strategies have been proposed.

#### Open-access curated database

To advance research in the field, it is important to provide practitioners and researchers with a ready-made database of selected knowledge. Current web resources are highlighted in Table 5. However, such databases are scarce, which can lead to valuable time being wasted and hinder research efforts. Therefore, it is necessary to establish new databases or contribute to existing ones as a member of a volunteer team, a crowdsourcing participant, or a crowdfunding supporter. In particular, it is important to link the microbiota reference genome databases, the microbiota-diseases relationships databases, and the microbiota-drug association prediction databases to the pharmacomicrobiomic data resource.

**Table 5:** Database for pharmacomicrobiomics studies

Category	Abbreviations	PMID
Microbiota reference genome	SILVA	17947321
	RDP	24288368
	NCBI-RefSeq	24316578
	MG-RAST	26656948
	MGnify	31696235
	IGC	24997786
	UHGG	32690973
Microbiota and disease relationships	HMDAD	26883326
	Disbiome	29866037
	gutMDisorder	31584099
	KATZHMDA	28025197
	BMCMDA	30367598
	LGRSH	32351464
Microbiota-drugs associations prediction	PharmacoMicrobiomics database	29652572
	MDAD	30581775
	RapidAIM	32160905
	GCNMDA	32597948

#### Techniques and approaches

**Fig. 5:**
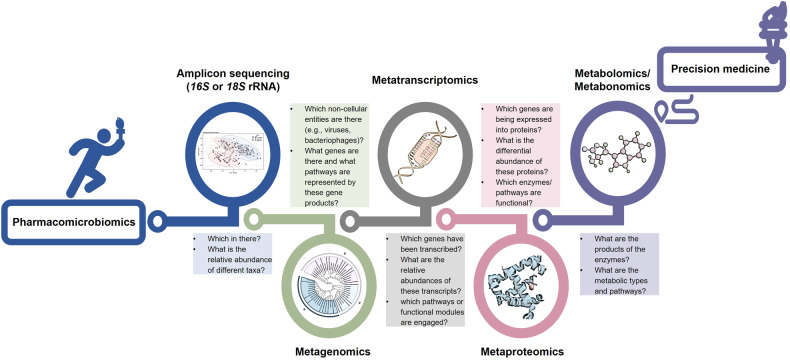
Multi-omics strategies facilitating pharmacomicrobiomics towards human precision medicine

The methods for investigating pharmacomicrobiomics are identical to the overall techniques employed to decipher the microbiota at various levels, including its taxonomic and genomic makeup, gene inventory, functional capacity, the tangible manifestation of its genes (at the RNA or protein level), and ultimately, its true functionality is indicated by the amalgamation of metabolites derived from the microbiota. Therefore, the analysis of drug-microbiota interactions can be effectively conducted using various techniques including amplicon sequencing (*16* *S* or *18* *S* rRNA subunit), shotgun microbiota sequencing or metagenomics, metatranscriptomics, metaproteomics, as well as metabolomics and metabonomics, as listed in Fig. 5.

These high-throughput data need to be modeled by artificial intelligence (AI) algorithms using advanced computational techniques.^492^ Machine learning is a sub-field of AI in which machines can acquire knowledge from data without the need for explicit programming.^199^ Machine learning includes both supervised and unsupervised algorithms. Classification or prediction tasks often use supervised algorithms, while clustering tasks often use unsupervised algorithms to group data based on similarity. The application of these methods could effectively address the needs of clustering and predicting patients, as well as discovering new biomarkers.^493^ The integrated analysis of multi-omics data has been enhanced by advances in machine learning. These approaches consist mainly of concatenation, modeling, and transformation-based techniques.

#### Predictive model and software engineering

It is essential to develop predictive models and software that simulate drug-microbiota interactions to reduce research time and costs. A major limitation in developing such models is the need for aggregated evidence to drive reliable assumptions that can be applied for testing and validation. By combining chemoinformatics with genomic and proteomic similarities, new interactions between drugs and various microbial enzymes can be predicted. This would significantly facilitate the establishment of predictive models for drug-microbiota interactions.

#### Prebiotics and probiotics

Prebiotics, which are indigestible components of food, stimulate the development of beneficial microbiota in the gut, while probiotics are live microorganisms that can be beneficial to health when ingested in moderation.^494^ Both prebiotics and probiotics have the potential to modify the composition and activity of gut microbiota and potentially enhance drug efficacy and reduce ADRs.^495^ Several recognized effects include: anti-bacterial effects by modulating pathogens’ genetic code composition, inhibiting competition for pathogens’ binding domains, inhibiting pathogens’ virus code or proteome composition, and stimulating the immune response by increasing anti-inflammatory cytokines and rescuing and modulating pro-inflammatory compounds.^495^

#### Fecal microbiota transplantation

FMT involves the transfer of fecal material from healthy donors to patients to restore gut microbiota.^496^ Initially, FMT was developed to treat *C. difficile* infection, more recently it has been highlighted as a promising approach to treating some complex digestive diseases.^497,498^ In addition to providing a diverse, stable gut microbiota, FMT also provides AAs, SCFAs, and BAs that assist in restoring normal gut physiological activity.^499^ Furthermore, the donor microbiota and their related anti-bacterial products, like adhesins, may be able to competitively match the site, inhibiting pathogenic microbiota colonization of the gut and preventing damage, which is a key action of FMT treatment.^500^

#### Bacteriophage therapy

Bacteriophage therapy, a practice dating back to the 1940s, utilizes naturally-occurring viruses to combat bacterial infections but has recently been enhanced with bio-engineered phages and associated lysing proteins to fight multi-drug resistant microbiota.^501^ Phages infect and lyse bacteria, and have several advantages, such as minimal harm to humans, breakdown of biofilms, specificity to certain hosts, and self-replication.^502–504^ Additionally, phages play a crucial role in ecosystem regulation by controlling microbiota.^505–507^ Phage therapy involves the use of ‘phage cocktail’, a mixture of lytic phages proven effective against the targeted pathogen.^502^ However, there are challenges associated with the therapy, including the need to selectively induce lysogenic phages, persistence in the gut, and immune responses.^508,509^ Phage therapy must also avoid interfering with standard microbiota function and creating selective pressure on non-target microbiota, which can lead to resistant offspring and disrupt beneficial bacteria and host adaptation mechanisms.^510^ The field is still expanding and requires further exploration.

## Conclusions and perspectives

### Microbiota biomarkers as promising targets in precision medicine

Human microbiota are valuable resources for identifying targets associated with various illnesses and IVDR. These biomarkers could potentially be used as effective replacements for procedures such as biomicroscopy and endoscopy, making them a promising tool for diagnosis and prediction.^43,511^ The microbiota releases substances and metabolites that can influence the occurrence, development, and outcome of disease, which highlights the value of microbiota biomarkers for individualized treatment.^512,513^ However, the inter-individual variability of the human microbiota, the emergence of new multi-drug resistance microbiota strains, and different microbiota drugs modify mechanisms hamper precision medicine. Nevertheless, precision medicine still holds the ideal opportunity for future theranostics, with a full understanding of the impact of the microbiota on IVDR allowing stratifying patients on the basis of identified biomarkers, microbiota types, and metabotypes.^514,515^

### Multi-omics enabling pharmacomicrobiomics

The integration of multiple high-throughput multi-omics datasets can provide a comprehensive profile of IVDR, both at a populational and single-cell level. The database includes five modules: amplicon sequencing (*16* *S* or *18* *S* rRNA subunit), metagenomics, metatranscriptomics, metaproteomics, and Metabolomics/Metabonomics (Fig. 5). This approach allows the investigation into the genetic variation and the regulation of molecular pathways in diseases and facilitates novel therapeutic strategies. To support extensive IVDR research, an open and integrated database, called the IVDR Atlas, has been established. These modules provide researchers with the opportunity to explore extensive gene variation and modulation datasets generated by multi-omics sequencing.

### From the lab to the clinical practice

Hopefully, the nascent discipline of pharmacomicrobiomics, which involves the study of drug-microbiota and drug-microenzyme interactions, is gaining traction due to the accessibility of on-line information, biomarker identification, and advances in methods, models, and software. The continuation of this pattern is anticipated through increased systematic screening, which will aid in the creation of new clinical practice recommendations and the implementation of existing guidelines. These developments, which are encouraged by the current advancements in technology, contribute to a growing tendency towards ‘precision medicine’ and the ‘One Health approach’. It is anticipated that the inclusion of microbiota typing and pharmacomicrobiomic testing in treatment protocols and drug labels will become customary, although the timing of this paradigm change is difficult to forecast. However, it is clear that the field is moving towards precision medicine, which is increasingly favored from an experimental, ethical, and economic point of view.

If you have reached this point, we cordially invite you to participate in these endeavors. To achieve the dream of developing precision medicine based on a thorough understanding of the human microbiota and a diverse set of tools for manipulating it, a collaborative effort across multiple traditional disciplines is essential. However, the challenges in this field are enormous, and our current knowledge is limited. Therefore, without a significant increase in research efforts, we will not be able to realize the whole value of pharmacomicrobiomic.
